# Effects of Kang’ai injection combined with chemotherapy on immune function in advanced non-small cell lung cancer: a meta-analysis

**DOI:** 10.3389/fphar.2026.1743226

**Published:** 2026-01-23

**Authors:** Yaoyao Wang, Xiaomei Wang, Hongnian Zhang, Xin Zhao, Tingting Zhang, Xuemei Wang, Yanchun Wang

**Affiliations:** 1 Henan University of Chinese Medicine, Zhengzhou, Henan, China; 2 Zhengzhou University, Zhengzhou, Henan, China; 3 Henan Provincial People’s Hospital, Zhengzhou, Henan, China

**Keywords:** immune function, Kang’ai injection, meta-analysis, non-small cell lung cancer, platinum-based chemotherapy

## Abstract

**Background:**

Systematically evaluate the effects of Kang’ai Injection (KAI) combined with platinum-based chemotherapy on immune function, clinical efficacy, and safety in patients with advanced non-small cell lung cancer.

**Materials and methods:**

Relevant literature published from the inception of each database through September 2025 will be identified through systematic searches of Chinese and English electronic databases. Randomized controlled trials (RCTs) evaluating Kang’ai injection combined with chemotherapy for advanced non-small cell lung cancer will be screened against predefined inclusion and exclusion criteria. Two investigators will independently perform data extraction and quality assessment. Meta-analyses will be conducted using RevMan 5.3 and Stata 18.0 software. Publication bias will be assessed using funnel plots and Egger’s test, while the robustness of findings will be examined through trial sequential analysis (TSA). The quality of evidence for critical outcomes will be evaluated using the GRADE approach.

**Results:**

A total of 14 randomized controlled trials involving 1,214 patients were included. The meta-analysis demonstrated that compared with chemotherapy alone, KAI combined with chemotherapy significantly improved the objective response rate and enhanced immune function parameters, including increased CD3^+^ and CD4^+^ T-cell counts, elevated CD4^+^/CD8^+^ ratio, and higher natural killer cell percentage, while reducing CD8^+^ T-cell percentage. The combination therapy group also showed superior outcomes in reducing tumor marker and vascular endothelial growth factor levels compared to the chemotherapy-alone group. Furthermore, combination treatment significantly reduced the incidence of chemotherapy-related adverse reactions including leukopenia, myelosuppression, nausea and vomiting, and gastrointestinal reactions.

**Conclusion:**

As an adjunctive therapy, KAI can enhance immune function (low-quality evidence), improve the objective response rate to chemotherapy (moderate-quality evidence), and alleviate chemotherapy-related toxicities (predominantly moderate-quality evidence) in patients with advanced NSCLC, providing an evidence-based reference for comprehensive clinical management.

**Systematic Review Registration:**

https://www.crd.york.ac.uk/PROSPERO/view/CRD420251168090.

## 1 Introduction

Lung cancer remains one of the most prevalent malignancies globally. Epidemiological investigations indicate that it ranks first among the eight major cancers in terms of new cases, accounting for 12.4% of all cancer cases worldwide. Furthermore, it represents the most frequently diagnosed cancer among male populations (Bray et al., 2024). Epidemiological studies have identified tobacco smoking as the primary risk factor for lung cancer pathogenesis, with additional contributions from environmental and occupational exposures, chronic pulmonary diseases, pulmonary infections, and lifestyle factors (Bade and Dela Cruz, 2020). Lung cancer is histologically classified into small cell lung cancer (SCLC) and non-small cell lung cancer (NSCLC), with NSCLC comprising approximately 85%–90% of all diagnosed cases (Ernani et al., 2017). For patients with early-stage NSCLC, surgical resection represents the optimal therapeutic approach (Hoy et al., 2019). However, for the majority of patients with advanced NSCLC, chemotherapy, immune checkpoint inhibitors, and targeted therapies constitute the mainstay of treatment (Li et al., 2023). The most frequently employed therapeutic regimens include PEB (cisplatin, etoposide, bleomycin), carboplatin monotherapy (AUC 7), PEI (cisplatin, etoposide, ifosfamide), TIP (cisplatin, ifosfamide, paclitaxel), and GOP (gemcitabine, oxaliplatin, paclitaxel). However, these treatment modalities are frequently associated with significant toxicities, including anemia, neutropenia, nausea, vomiting, and diarrhea (Zraik and Heß-Busch, 2021). These treatment-related toxicities not only exacerbate patients’ pain but also contribute to depression and other psychological and social distress. Furthermore, they can compromise the immune system of NSCLC patients and significantly impair their quality of life (De Ruysscher et al., 2020). Therefore, enhancing immune function is crucial for prolonging survival and improving quality of life in patients with advanced NSCLC.

The most basic theory of TCM in the treatment of NSCLC is regulating the imbalance by “strengthening the body” and “eliminating evil” when the body’s immunity is too weak and the tumor growth ability is too strong. “Strengthening the body” means enhancing the body’s anti-cancer immunity, while “ eliminating evil ” directly inhibits tumor cell growth, proliferation, invasion, and migration (Li et al., 2021).

Integrated Chinese-Western medicine therapy has demonstrated promising outcomes in the management of NSCLC. This approach enhances sensitivity to chemotherapy and radiotherapy while reducing treatment-related adverse effects, including myelosuppression, nausea, and vomiting, as well as other associated complications. Kang’ai Injection (KAI) is a standardized botanical drug preparation approved by the China National Medical Products Administration (NMPA; Approval Number: Z20026868) and manufactured by Changchun Baishan Pharmaceutical Co., Ltd. According to the product specification, it is composed of the following substances: *Panax ginseng* C.A. Mey. [Araliaceae; GINSENG RADIX ET RHIZOMA] (Renshen)、*Astragalus membranaceus* (Fisch.) Bunge [Fabaceae; ASTRAGALI RADIX] (Huangqi)、*Sophora flavescens* Aiton [Fabaceae; SOPHORAE FLAVESCENTIS RADIX] (Kushen), all plant materials were verified against the Plants of the World Online (POWO) database on 7 November 2025. The sourcing, processing, and production of KAI utilized in this research were conducted in full compliance with the Nagoya Protocol and relevant Chinese plant quarantine regulations (Greiber, 2019) ts preparation process focuses on the targeted extraction of active components and multi-step purification: In the extraction stage, differentiated protocols are adopted based on the characteristics of different raw materials. For Sophora flavescens (Kushen), 0.5% acetic acid aqueous solution is used as the solvent, and ultrasonic/heating reflux extraction is conducted at 50 °C–80 °C (1–2 h per time, repeated 2–3 times) to enrich alkaloids such as oxymatrine. For Panax ginseng (Renshen), 50%–70% ethanol aqueous solution is employed for heating reflux extraction at 80 °C–85 °C (1.5–2 h per time, repeated twice) to obtain saponins including ginsenoside Rg1 and Re. For Astragalus membranaceus (Huangqi), purified water is used at a solid-liquid ratio of 1:10–1:12 for decoction at 95 °C–100 °C (1.5–2 h per time, repeated twice) to retain saponins such as astragaloside IV. In the purification stage, macromolecular impurities like proteins and polysaccharides are first removed via ethanol precipitation with 70%–80% ethanol (standing at 4 °C for 12–24 h followed by centrifugation at 8000 rpm for 5 min). Subsequently, D101/HPD-100 type macroporous resins are used to enrich ginsenosides (from Panax ginseng) and astragalosides (from Astragalus membranaceus) through water elution for impurity removal and 50%–70% ethanol elution, while 732 type cation exchange resins are applied to purify alkaloids from Sophora flavescens. Finally, ultrafiltration with a 10 kDa ultrafiltration membrane and autoclaving at 121 °C for 15 min are performed to produce a sterile, pyrogen-free intravenous injection, ensuring a high recovery rate of target components throughout the process (Yuan et al., 2017; Jia et al., 2020). The combination of botanical drug formulations with chemotherapy has been demonstrated to reduce the toxicity associated with adjunctive chemotherapy (Wang et al., 2020). In addition to reducing the incidence of adverse reactions, the combination of KAI with platinum-based chemotherapy demonstrates enhanced clinical efficacy and possesses immunomodulatory properties within the tumor microenvironment (Li et al., 2019). Furthermore, Kang’ai injection has been utilized as an adjunctive therapy for various malignancies, demonstrating potential in suppressing hepatocellular carcinoma cell proliferation (Sun et al., 2021)、nasopharyngeal carcinoma cells (Wang et al., 2025) and colorectal cancer (Gao and Zhang, 2023) including nausea and vomiting, hepatic impairment, peripheral neurotoxicity, pyrexia, abdominal pain, alopecia, elevated bilirubin levels, and leukopenia induced by conventional therapies. Although KAI has demonstrated the ability to reduce toxic side effects and improve immune function as an adjunctive therapy for advanced NSCLC, current studies regarding its efficacy and safety in cancer treatment exhibit heterogeneity in sample size and research design. Therefore, we conducted this updated meta-analysis in accordance with the PRISMA checklist, aiming to comprehensively evaluate the efficacy and safety of KAI combined with conventional therapy in advanced NSCLC, thereby providing evidence for clinical practice.

## 2 Materials and methods

### 2.1 Protocols and registration

This systematic review and meta-analysis followed the methodological guidelines of the Preferred Reporting Items for Systematic Reviews and Meta-Analyses (PRISMA) (Page et al., 2021) to ensure methodological rigor. Moreover, the research protocol was prospectively registered with the International Prospective Register of Systematic Reviews (PROSPERO) under registration number CRD420251168090.

### 2.2 Literature search strategy

A comprehensive literature search was conducted utilizing the following electronic databases from their inception through September 2025: English databases including PubMed, the Cochrane Library, Web of Science, and Embase; Chinese databases including CNKI, Wanfang, VIP, CBM and the CMJD. The search strategy incorporated both Medical Subject Headings (MeSH) and free-text terms to identify all relevant studies. For English databases, the following search strategy was implemented: (Neoplasm [Mesh] OR Lung Neoplasm [Mesh] OR Pulmonary Neoplasms OR Lung Cancer OR NSCLC OR Non-small Cell Lung Cancer) AND (Chemotherapy OR Chemotherapeutics OR Chemical therapy) AND (Kangai injection OR Kangai OR Kang’ai). For Chinese databases, the following search strategy was adopted: [Kangai injection] AND [Chemotherapy] AND [Non-small Cell Lung Cancer].

### 2.3 Inclusion criteria

The inclusion criteria were as follows: (1) Study Type: Randomized Controlled Trial (RCT), which must clearly report the method of random sequence gen1eration (e.g., random number table, computer randomization, etc.), with traceable methodology. (2) Study Subjects: Patients with a histopathological or cytopathological diagnosis of advanced non-small cell lung cancer (NSCLC, TNM stage III-IV). Age and gender are unrestricted, and baseline data (e.g., age, gender, tumor stage) must be complete. (3) Interventions: The experimental group receives Kang’ai Injection (KAI) combined with a platinum-based chemotherapy regimen. The control group receives only the same platinum-based chemotherapy regimen as the experimental group. The dosage and treatment cycles of chemotherapeutic drugs are identical for both groups. (4) Outcome Measures: The study must report at least one of the following core outcomes: ① Immune function indicators (one of CD3^+^ T cells, CD4^+^ T cells, CD8^+^ T cells, CD4^+^/CD8^+^ ratio, NK cell percentage); ② Objective Response Rate (ORR); ③ Chemotherapy-related adverse reactions (one of leukopenia, bone marrow suppression, nausea and vomiting, gastrointestinal reactions). (5) Methodological Quality: Among the methodological quality assessment items for included studies, the three core items “random sequence generation,” “allocation concealment,” and “selective reporting” must not be labeled as “high risk.” Studies with vague randomization methods or illogical grouping (e.g., grouping by treatment regimen or order of presentation) are excluded. (6) Concomitant Medications: Patients must not be concurrently receiving concomitant medications that may affect immune function or tumor progression, such as PD-1/PD-L1 inhibitors, immunomodulators, or other traditional Chinese medicine injections. Only symptomatic supportive treatments (e.g., antiemetics, fluid replacement, nutritional support) are allowed, and the supportive treatment regimen must be consistent between the two groups.

### 2.4 Exclusion criteria

The exclusion criteria were as follows: (1) Non-RCT Studies: Such as cohort studies, case-control studies, cross-sectional studies, animal experiments, *in vitro* cell experiments, etc. (2) Non-Eligible Study Subjects: Patients with non-advanced NSCLC (TNM stage I-II), or those with underlying conditions that may affect outcome assessment, such as other malignant tumors, severe hepatic or renal insufficiency, autoimmune diseases, or severe infections. (3) Non-Compliant Interventions: The experimental group combined other anti-tumor drugs, immunotherapy, radiotherapy, etc.; the control group’s chemotherapy regimen differed from that of the experimental group; or the dosage or treatment course of Kang’ai Injection was unclear. (4) Missing or Incomplete Outcome Measures: Core immune function indicators, ORR, or adverse reaction data were not reported, or effective statistics (e.g., mean, standard deviation, sample size, effect size, etc.) could not be extracted. (5) Serious Methodological Flaws: Clearly identified as having a “high risk” of bias (e.g., incorrect random sequence generation, selective outcome reporting, data fabrication, etc.); duplicate publications (the study with the larger sample size and more complete data will be prioritized for inclusion). (6) Interference from Concomitant Medications: Patients received immune-related drugs during treatment (e.g., PD-1/PD-L1 inhibitors, CTLA-4 inhibitors, immunoglobulins, cytokines) or concurrently used other traditional Chinese medicine compounds or Chinese patent medicines with anti-tumor effects, which may interfere with outcome judgment. (7) Non-Eligible Publication Types: Non-original research such as reviews, meta-analyses, case reports, conference abstracts, or dissertations (unpublished and with data not publicly verified). Studies meeting any of the above criteria will be manually excluded.

### 2.5 Outcome measures

The primary outcome measures were objective response rate (ORR) and immune function indicators, while the secondary outcomes included adverse reactions, cytokines, and tumor markers: ① The objective response rate was determined in accordance with the World Health Organization (WHO) Response Evaluation Criteria in Solid Tumors (RECIST). RECIST classifies tumor response into complete response (CR), partial response (PR), stable disease (SD), and progressive disease (PD). he calculation formula is: ORR = (CR + PR)/Total number of cases × 100%. ② Immune function indicators included peripheral blood T lymphocyte subsets (CD3^+^, CD4^+^, CD8^+^, CD4^+^/CD8^+^) and natural killer (NK) cell ratio. ③ Adverse reactions were classified into grades 0-IV based on the acute and subacute toxicity grading criteria specified by the WHO, with grades II-IV considered as the presence of adverse reactions. The outcomes of adverse reactions included leukocyte toxicity, nausea and vomiting toxicity, myelosuppression toxicity, and gastrointestinal reaction toxicity. ④ Cytokine outcomes included the level of vascular endothelial growth factor (VEGF), which is used to assess tumor angiogenesis activity; tumor marker outcomes included carcinoembryonic antigen (CEA), which is used to evaluate tumor burden and treatment response.

### 2.6 Data extraction and quality assessment

Two researchers independently screened and summarized the collected literature, extracted relevant information, and then conducted cross-verification. Any discrepancies were resolved through discussion or with the assistance of a third researcher. The extracted content included: ① title, first author, and year of publication; ② number of patients, age, gender, and TNM staging in the experimental group and control group; ③ specific intervention measures, outcome indicators, etc. The two researchers also assessed the methodological quality of all randomized controlled trials (RCTs) in accordance with the risk of bias criteria outlined in the Cochrane Handbook Version 6.2. The assessment items included: random sequence generation, allocation concealment, blinding, incomplete data assessment, selective outcome reporting, and other potential biases. Each of these items was evaluated using one of three responses: “Yes”, “No”, or “Unclear”. In addition, the two researchers applied the GRADE ProGuideline Development Tool based on the published protocol (Terracciano et al., 2010) to assess the quality of each outcome. The evidence quality assessment is categorized into four levels: high quality (High), moderate quality (Moderate), low quality (Low), and very low quality (Very Low).

### 2.7 Statistical analysis

For the meta-analysis, RevMan 5.3 software (Cochrane Collaboration) and Stata 18.0 software were used. Relative Risk (RR) was applied for dichotomous variables, while Mean Difference (MD) was used for continuous variables. Both were calculated with a 95% Confidence Interval (CI). I^2^ statistic was employed to quantitatively assess heterogeneity. I^2^ > 50%, heterogeneity was considered high, and a random-effects model was adopted. I^2^ < 50%, heterogeneity was deemed low, and a fixed-effects model was used. When the meta-analysis included more than 10 studies, a funnel plot was used to evaluate publication bias. Additionally, Egger’s test was performed using Stata 18.0 software to assess the asymmetry of the funnel plot. Trial sequential analysis (TSA) was performed using TSA software version 0.9.5.10 Beta to evaluate the robustness of the findings and calculate the required information size for definitive conclusions.

## 3 Results

### 3.1 Retrieval results

**FIGURE 1:**
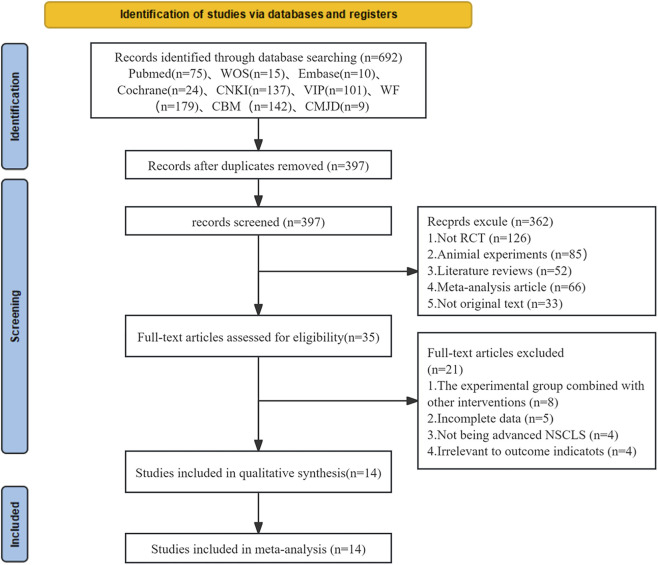
Flowchart of literature search and screening.

A total of 692 studies were initially identified, of which 14 met the predefined inclusion criteria as illustrated in Figure 1 (Chen, 2014; Ma, 2017; Wang, 2017; Gao, 2018; Jiang et al., 2018; Dong et al., 2019; Tang, 2019; Xing, 2020; Wu, 2021; Cheng, 2022; Wu et al., 2022a; Xue et al., 2022; Gong et al., 2024; Nie and Xiong, 2025). Based on the predetermined inclusion and exclusion criteria, a total of 1,214 patients were ultimately included in the final analysis.

### 3.2 Characteristics included in the study

Tables 1,2 list all the baseline characteristics of the included studies, including author, publication year, number of cases, TMN stage, gender, age, details of intervention, treatment duration, and outcomes. All studies were conducted in China, with publication years ranging from 2014 to 2025. A total of 14 randomized controlled trials were included, enrolling 1,214 patients, with 634 in the experimental group and 580 in the control group. All included non-small cell lung cancer cases were at TNM stage III-IV. The control group received first-line platinum-based chemotherapy regimens, including NP, GP, PC, Bev + PEM + DDP, TP, DP + Bev, PF, TP, and DDP. The intervention in the treatment group was the combination of KAI (intravenous drip, once daily, 10–60 mL, with at least 10 days as one course) on the basis of the control group. The study results showed that all studies reported ORR and immune function indicators. Among them, 13 studies reported CD3^+^, 14 studies reported CD4^+^, 12 studies reported CD8^+^, 14 studies reported CD4^+^/CD8^+^, 4 studies reported NK cell percentage, 5 studies reported data on CEA,6 studies reported leukotoxicity, 8 studies reported myelosuppression toxicity, 6 studies reported nausea and vomiting toxicity, and 5 studies reported gastrointestinal reaction toxicity.

**TABLE 1:** Baseline characteristics of the included studies.

Study	Cases	Stage	Gender (M/F)		Age/Y		Intervention		No.	Outcome
​	T/C	​	T	C	T	C	T	C	​	​
Chen. (2014)	41/42	III_a_-IV	28/13	25/17	62.4 ± 10.5	61.9 ± 10.7	KAI + NP (40 mL/d,d1-d21)	NP	2	①②
Cheng. (2022)	44/44	III_b_-IV	25/19	27/19	40–75 (57.13 ± 6.15)	40–73 (56.89 ± 5.94)	KAI + GP (40 mL/d,d1-d21)	GP	2	①②⑤⑥⑦
Dong et al. (2019)	56/56	III_a_-IV	35/21	37/19	66.12 ± 3.45	65.74 ± 3.52	KAI + GP (40 mL/d,d1-d14)	GP	4	①②③④
Gao (2018)	29/29	III_b_-IV	17/12	17/12	34–77 (53.82 ± 7.65)	34–76 (54.15 ± 7.26)	KAI + PC (40 mL/d,d1-d21)	PC	2	①②③⑤⑥⑧
Gong et al. (2024)	30/30	III-IV	19/11	18/12	38–76 (45.89 ± 5.12)	36–77 (45.65 ± 5.65)	KAI + Bev + PEM + DDP (10 mL/d,d1-d21)	Bev + PEM + DDP	2	①②⑤⑧
Jiang et al. (2018)	43/42	III_b_-IV	26/17	26/16	58.85 ± 10.16	58.34 ± 10.42	KAI + GP (50 mL/d,d1-d14)	GP	4	①②⑤⑦
Ma. (2017)	40/42	III_a_-IV	26/14	27/15	48–77 (57.08 ± 6.43)	46–75 (57.43 ± 6.76)	KAI + TP (40 mL/d,d1-d21)	TP	4	①②④⑥⑧
Nie and Xiong. (2025)	44/44	III-IV	29/15	27/17	60.86 ± 6.27	60.41 ± 6.18	KAI + TP (60 mL/d,d1-d21)	TP	3	①②③⑥⑧
Tang. (2019)	32/28	III_b_-IV	20/12	18/10	46–76 (60.98 ± 5.35)	47–77 (61.56 ± 6.43)	KAI + DP + Bev (50 mL/d,d1-d21)	DP + Bev	2	①②④⑥⑦
Wang (2017)	38/38	IV	26/12	28/10	22–73 (56.9 ± 14.1)	21–74 (57.2 ± 14.4)	KAI + PF (40 mL/d,d1-d21)	PF	1	①②⑥⑦
Wu et al. (2022b)	50/50	III-IV	31/19	29/21	66.08 ± 4.03	65.17 ± 3.20	KAI + TP (60 mL/d,d1-d21)	TP	2	①②④⑤⑥⑧
Wu (2021)	37/35	III_b_-IV	21/16	23/12	41–88 (62.6 ± 5.7)	40–86 (62.8 ± 5.5)	KAI + GP (50 mL/d,d1-d14)	GP	4	①②⑤⑦
Xing (2020)	50/50	III_a_-IV	26/24	3/27	38–73 (57.3 ± 5.8)	37–72 (57.0 ± 6.3)	KAI + DDP (50 mL/d,d1-d10)	DDP	2	①②③④
Xue et al. (2022)	100/50	III_a_-IV	67/33	32/18	65–81 (60.27 ± 3.95)	64–83 (61.31 ± 4.07)	KAI + GP (40 mL/d,d1-d14)	GP	4	①②③④⑥⑦

KAI, Kang’ai injection; T, treatment; C, control; M, male; F, female; Y, year; No, Number of KAI, cycles. NP, Vinorelbine + Platinum; GP, Gemcitabine + Platinum; PC, Pemetrexed + Cisplatin; Bev + PEM + DDP, Bevacizuma + Pemetrexed + Cisplatin; TP, Paclitaxel + Cisplatin; DP + Bev, Docetaxel + Cisplatin + Bevacizumab; PF, Cisplatin + Fluorouracil; TP, Paclitaxel + Cisplatin; DDP, cisplatin; ①ORR; ②Immune function; ③CEA; ④VEGF; ⑤ Leukocytotoxicity⑥ Myelosuppressive toxicity⑦ Nausea and vomiting toxicity⑧ Gastrointestinal reaction toxicity.

**TABLE 2:** Specific immune function indicators of the included studies.

Study	Immune function
Chen. (2014)	CD3^+^、CD4^+^、CD8^+^、CD4^+^/CD8^+^
Cheng. (2022)	CD3^+^、CD4^+^、CD8^+^、CD4^+^/CD8^+^、NK
Dong et al. (2019)	CD3^+^、CD4^+^、CD8^+^、CD4^+^/CD8^+^
Gao. (2018)	CD3^+^、CD4^+^、CD8^+^、CD4^+^/CD8^+^
Gong et al. (2024)	CD3^+^、CD4^+^、CD4^+^/CD8^+^
Jiang et al. (2018)	CD3^+^、CD4^+^、CD4^+^/CD8^+^、NK
Ma. (2017)	CD3^+^、CD4^+^、CD4^+^/CD8+
Nie and Xiong. (2025)	CD4^+^、CD8^+^、CD4^+^/CD8^+^
Tang. (2019)	CD3^+^、CD4^+^、CD8^+^、CD4^+^/CD8^+^
Wang. (2017)	CD3^+^、CD4^+^、CD8^+^、CD4^+^/CD8^+^、NK
Wu et al. (2022b)	CD3^+^、CD4^+^、CD8^+^、CD4^+^/CD8^+^
Wu. (2021)	CD3^+^、CD4^+^、CD8^+^、CD4^+^/CD8^+^、NK
Xing. (2020)	CD3^+^、CD4^+^、CD8^+^、CD4^+^/CD8^+^
Xue et al. (2022)	CD3^+^、CD4^+^、CD8^+^、CD4^+^/CD8^+^

### 3.3 Methodological quality assessment

**FIGURE 2:**
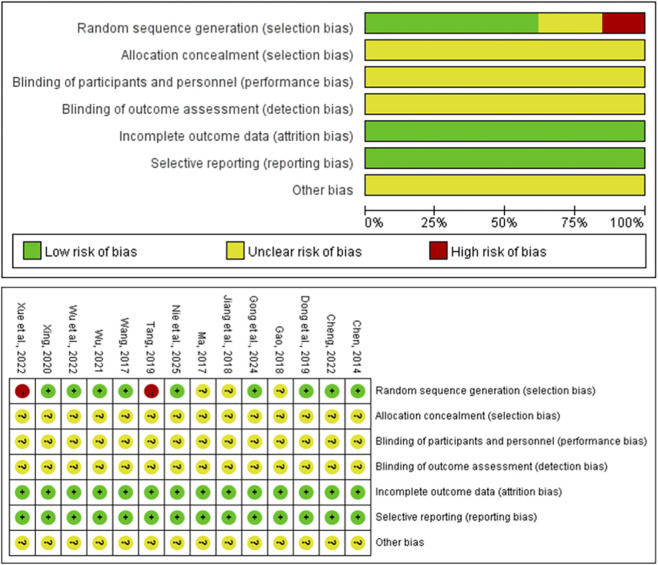
Bias chart of the included studies.

Eight studies using a table of random numbers were rated as ‘low risk’. Six studies mentioned the term ‘random’ but did not specify the exact method, and even after contacting by email, it remained unclear, so they were rated as ‘unclear’. Three studies used incorrect random sequence generation, such as grouping according to treatment plans. In 14 studies, it was not possible to determine whether allocation concealment was used, so they were rated as ‘unclear’. No study mentioned a ‘blinding’ strategy, so they were rated as ‘unclear’. No study reported participant dropout, so they were rated as ‘low risk’. No study mentioned selective reporting, so they were rated as ‘low risk’. In all studies, it could not be determined whether there were other biases, so they were all rated as ‘unclear’ (Figure 2).

### 3.4 Outcome indicators

#### 3.4.1 Objective response rate (ORR)

**FIGURE 3:**
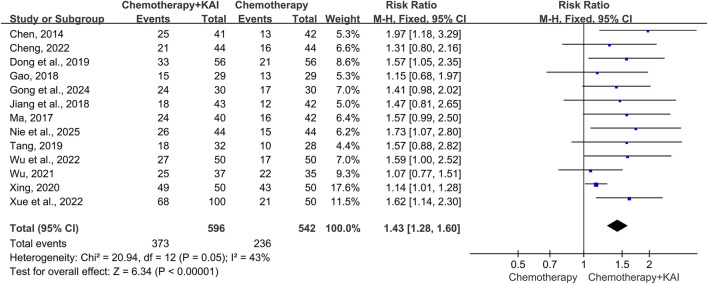
Meta-analysis of objective response rate (ORR).

Thirteen studies (n = 1,138) reported ORR. Heterogeneity analysis indicated an I^2^ = 43% (P = 0.05), and a fixed-effect model was employed. The results showed that the ORR in the KAI combined with chemotherapy group was significantly higher than that in the chemotherapy-alone group [RR = 1.43, 95% CI (1.28, 1.60), P < 0.00001] (Figure 3).

#### 3.4.2 Immune function indicators

**FIGURE 4:**
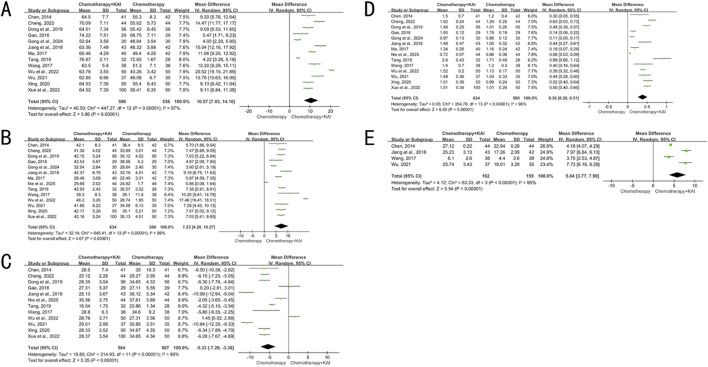
**(A)** meta-analysis of CD3^+^. **(B)** Meta-analysis of CD4^+^. **(C)** Meta-analysis of CD8^+^. **(D)** meta-analysis of CD4^+^/CD8^+^. **(E)** meta-analysis of NK.

Thirteen studies (n = 1,126) analyzed CD3^+^ T cells, fourteen studies (n = 1,214) analyzed CD4^+^ T cells and the CD4^+^/CD8^+^ ratio, twelve studies (n = 1,071) analyzed CD8^+^ T cells, and four studies (n = 321) analyzed NK cells. High heterogeneity was present for all analyses (I^2^ = 95%–98%, P < 0.00001), and a random-effects model was employed. The results showed that the combination therapy group had significantly increased proportions of CD3^+^ and CD4^+^ T cells, a higher CD4^+^/CD8^+^ ratio, and a higher percentage of NK cells (MD = 10.57, 7.23, 0.38 and 5.84, respectively; 95% CIs did not cross 0; P < 0.00001 for all). The proportion of CD8^+^ T cells was significantly decreased [MD = −5.33, 95% CI (−7.29, −3.38), P < 0.00001] (Figure 4).

#### 3.4.3 Tumor markers and cytokines

**FIGURE 5:**
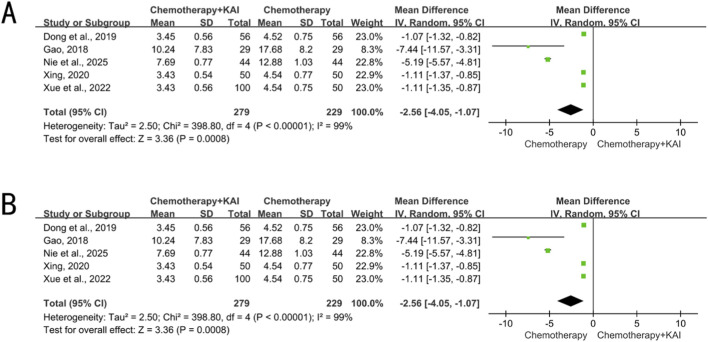
**(A)** meta-analysis of CEA. **(B)** Meta-analysis of VEGF.

Five studies (n = 508) analyzed CEA (I^2^ = 99%, P < 0.00001) and six studies (n = 604) analyzed VEGF (I^2^ = 33%, P = 0.19). The levels of both CEA [MD = −2.56, 95% CI (−4.05, −1.07), P = 0.0008] and VEGF [MD = −34.43, 95% CI (−39.29, −29.57), P < 0.00001] were significantly lower in the combination therapy group compared to the chemotherapy-alone group (Figure 5).

#### 3.4.4 Reduction of toxicity

**FIGURE 6:**
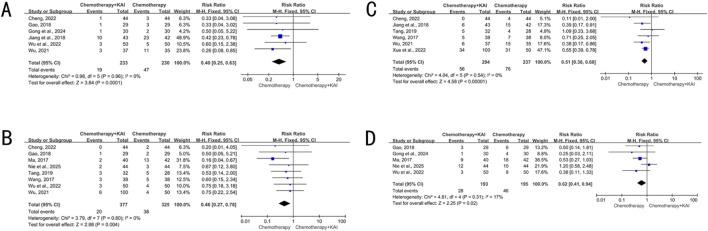
**(A)** Meta-analysis of leukopenia. **(B)** Meta-analysis of bone marrow suppression. **(C)** Meta-analysis of nausea and vomiting. **(D)** Meta-analysis of gastrointestinal reactions.

Six studies (n = 463) reported data on leukopenia (I^2^ = 0%, P = 0.96), eight studies (n = 702) reported on myelosuppression (I^2^ = 0%, P = 0.80), six studies (n = 531) reported on nausea/vomiting (I^2^ = 0%, P = 0.54), and five studies (n = 388) reported on gastrointestinal reactions (I^2^ = 17%, P = 0.31). A fixed-effect model was used for all analyses. The combination therapy group showed a significantly lower incidence of the aforementioned adverse reactions (RR = 0.40, 0.46, 0.51 and 0.62, respectively; 95% CIs did not include 1; P < 0.05 for all) (Figure 6).

### 3.5 Publication bias

**FIGURE 7:**
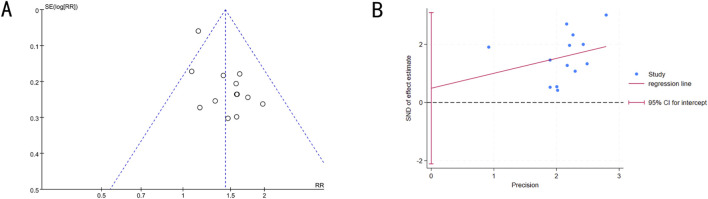
**(A)** Funnel plot of ORR. **(B)** Egger’s publication bias assessment plot.

Publication bias in studies reporting ORR was analyzed using funnel plots and Egger’s test. The funnel plot showed an asymmetric distribution, suggesting potential publication bias (Figure 7A). Therefore, Egger’s test was conducted for quantitative analysis (t = 0.42, 95%CI: −2.11027 to 3.09185, P = 0.686), and the results indicated no publication bias (Figure 7B).

### 3.6 Sensitivity analysis

**FIGURE 8:**
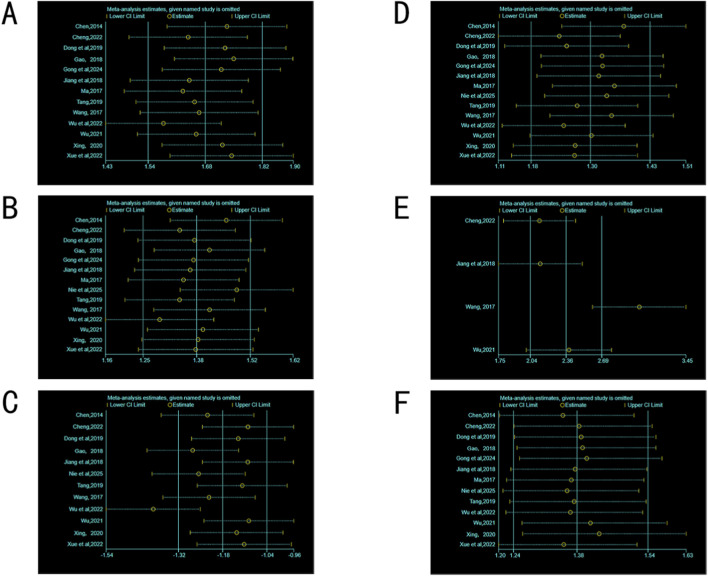
Sensitivity analysis. **(A)** (CD3^+^). **(B)** (CD4^+^). **(C)** (CD8^+^). **(D)** (CD4^+^/CD8^+^). **(E)** (NK). **(F)** (ORR).

As shown in Figure 8, the pooled MD of CD3^+^, CD4^+^, CD8^+^, CD4^+^/CD8^+^, NK cells, and ORR in the included studies was not significantly affected, and the 95% confidence interval (95%CI) did not cross the invalid line, indicating that the results were relatively stable.

### 3.7 Subgroup analysis

**FIGURE 9:**
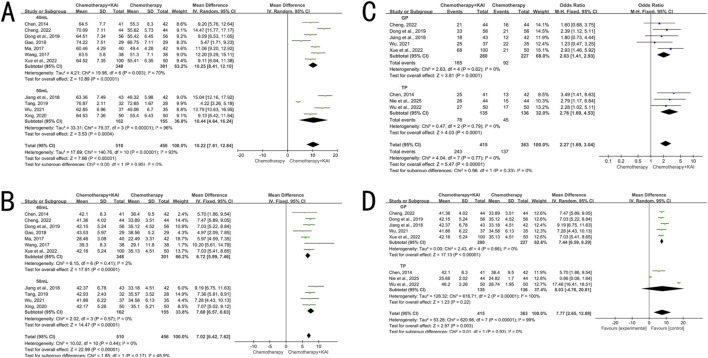
Subgroup analyses. **(A)** Dosage subgroup analysis of objective response rate (ORR); **(B)** Dosage subgroup analysis of CD4^+^; **(C)** ORR subgroup analysis by GP and TP regimens; **(D)** CD4^+^subgroup analysis by GP and TP regimens.

Subgroup analyses were performed for the ORR and CD4^+^ level, with stratification factors including KAI dosage (40 mL vs. 50 mL) and chemotherapy regimen (GP vs. TP). The results (Figure 9) showed that KAI combined with chemotherapy significantly improved ORR in all subgroups, with low heterogeneity within each subgroup. Tests for subgroup differences revealed no statistical heterogeneity between the two dosages, confirming the consistent efficacy of KAI in enhancing ORR at both 40 mL and 50 mL doses. In the GP and TP subgroups, ORR was also significantly improved with low within-subgroup heterogeneity. Additionally, subgroup difference tests indicated no statistical heterogeneity between the GP and TP regimens, suggesting consistent synergistic effects of KAI with both chemotherapy regimens in improving ORR. We speculate that this may be related to differences in baseline characteristics of included patients or treatment details.

### 3.8 Trial sequential analysis

**FIGURE 10:**
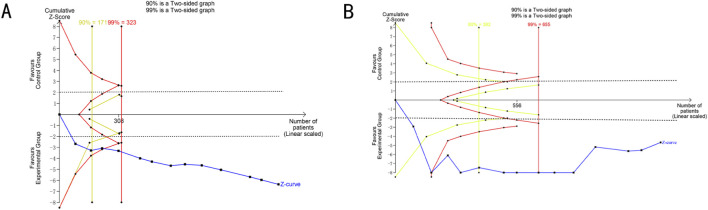
Trial sequential analysis. **(A)** ORR. **(B)** CD4^+^.

The TSA boundaries were used to assess the true effects of ORR and CD4^+^ and calculate the required sample size. For ORR, the type I error rate was set at 5%, the type II error rate at 20%, the relative risk reduction at 35%, and the incidence in the control group at 3%. For CD4^+^, the type I error rate was defined as 5%, and the power was set at 80%. The blue lines represent the TSA boundaries for ORR and CD4^+^. As shown in Figure 10, neither the cumulative Z boundary (blue line) nor the TSA boundary for CD4^+^ (blue line) exceeded the traditional Z boundary (green line) and the trial sequential monitoring boundary (red line), with the required sample size indicated by the vertical red line. The results showed that the total sample size included in this study met the requirements for meta-analysis, and the possibility of false positives could be excluded (Figure 10).

### 3.9 Quality of evidence

GRADE PRO GDT was used to assess the quality of all outcomes from five aspects, including risk of bias, inconsistency, indirectness, imprecision, and other considerations. The included studies had some deficiencies in randomization, allocation concealment, and blinding, and were rated as having a low level of risk of bias. The final results showed that four outcomes were of moderate quality and ten outcomes were of low quality, as shown in Tables 3,4.

**TABLE 3:** Summary of GRADE evidence for clinical efficacy and safety.

Outcomes (trials)	Quality assessment					Stage III/IV NSCLC		Clinical efficacy and safety		Quality
​	Risk of bias	Inconsistency	Indirectness	Imprecision	Publication bias	KAI + C	C	Relative ratio (95%CI)	Absolute (95%CI)	​
ORR (13)	Serious^a^	No	No	No	None	373/596 (62.6%)	236/54 (43.4%)	1.43 (1.28,1.60)	187 more per 1,000 (from 122 more to 260 more)	⊕⊕⊕○ Moderate
Leukocytotoxicity (6)	Serious^a^	Serious^b^	No	No	None	19/233 (8.2%)	47/230 (20.4%)	0.40 (0.25,0.63)	122 fewer per 1,000 (from 153 more to 75 more)	⊕⊕○○ low
Myelosuppressive toxicity (8)	Serious^a^	No	No	No	None	20/377 (5.3%	38/325 (11.7%)	0.64 (0.27,0.78)	63 fewer per 1,000 (from 85 more to 26 more)	⊕⊕⊕○ Moderate
Nausea and vomiting toxicity (6)	Serious^a^	No	No	No	None	56/294 (19.0%)	76/237 (32.1%)	0.51 (0.38,0.68)	157 fewer per 1,000 (from 199 more to 103 more)	⊕⊕⊕○ Moderate
Gastrointestinal reaction toxicity (5)	Serious^a^	No	No	No	None	28/193 (14.5%)	46/195 (23.6%)	0.62 (0.41,0.94)	90 fewer per 1,000 (from 139 more to 14 more)	⊕⊕⊕○ Moderate

ORR, objective response rate; CI, confidence intervals; KAI, Kang’ai injection; C, control.

^a^ The included studies have certain defects in randomization, allocation concealment and blinding.

^b^ The included studies are highly heterogeneous.

**TABLE 4:** Summary of GRADE evidence for immune indicators and tumor markers.

Indicators (Trials)	Quality assessment					Stage III/IV NSCLC		Clinical efficacy and safety		Quality
​	Risk of bias	Inconsistency	Indirectness	Imprecision	Publication bias	KAI + C	C	Relative ratio (95%CI)	WMD (95%CI)	​
CD3^+^(13)	Serious^a^	No	No	No	None	590	536	No	10.57 higher (7.03–14.10 higher)	⊕⊕○○ low
CD4^+^(14)	Serious^a^	No	No	No	None	634	580	No	7.23 higher (4.20–10.27 higher)	⊕⊕○○ low
CD8^+^(12)	Serious^a^	No	No	No	None	564	507	No	5.33 lower (7.29–3.38 lower)	⊕⊕⊕○ low
CD4^+^/CD8^+^(14)	Serious^a^	No	No	No	None	634	580	No	0.38 higher (0.26–0.51 higher)	⊕⊕○○ low
NK (4)	Serious^a^	No	No	No	None	162	159	No	5.84 higher (3.77–7.90 higher)	⊕⊕○○ low
CEA (5)	Serious^a^	No	No	No	None	279	229	No	2.65 lower (4.05–1.07 lower)	⊕⊕○○ low
VEGF (6)	Serious^a^	No	No	No	None	328	276	No	34.43 lower (39.29–29.57 lower)	⊕⊕○○ low

CD, cluster of differentiation; CI, confidence intervals; WMD, weighted mean difference; KAI, Kang’ai injection; C, control; NK, natural killer cell; CEA, carcinoembryonic antigen; VEGF, vascular endothelial growth factor.

^a^ The included studies have certain defects in randomization, allocation concealment and blinding.

## 4 Discussion

Although chemotherapy remains the first-line treatment for advanced NSCLC, its associated toxicities and side effects significantly compromise patients’ quality of life and immune function. While emerging evidence suggests potential benefits of KAI combined with chemotherapy in treating advanced NSCLC, comprehensive analyses of immune-related parameters and mitigation of treatment-related toxicities remain insufficient. Therefore, we conducted this updated meta-analysis to provide clinical guidance for advanced NSCLC management and preliminary evidence for fundamental research.

In this study, we selected a total of 14 randomized controlled trials involving 1,214 patients according to predefined criteria. The results demonstrated that KAI combined with chemotherapy significantly improved the objective response rate and enhanced immune function—particularly by increasing the CD4^+^/CD8^+^ ratio—while reducing chemotherapy-related toxicities such as myelosuppression and gastrointestinal reactions. The immune system plays a crucial role in controlling the development and progression of malignant tumors in humans (Lechner et al., 2017). In clinical practice, peripheral blood T lymphocyte subsets and NK cells have emerged as established biomarkers for monitoring tumor development and progression, as well as predicting therapeutic efficacy and prognosis (Meng et al., 2019; Peled et al., 2019). Therefore, CD3^+^, CD4^+^, and CD8^+^ T-cell counts, CD4^+^/CD8^+^ ratio, and NK cell percentage were selected as key indicators for evaluating immune function in NSCLC patients. The results demonstrated that compared with chemotherapy alone, the KAI combination therapy group showed significantly increased percentages of CD3^+^ and CD4^+^ T-cells, CD4^+^/CD8^+^ ratio, and NK cells, while exhibiting decreased CD8^+^ T-cell percentage, collectively indicating enhanced immune function in patients. The main subtypes of peripheral blood T lymphocyte subsets are CD3^+^, CD4^+^ and CD8^+^ cells. Among these, CD3^+^ T cells represent the total T lymphocyte population and reflect the functional status of the host cellular immunity. CD4^+^ T cells primarily function by secreting a broad spectrum of cytokines to promote immune responses, thereby exerting anti-tumor effects. The CD4^+^/CD8^+^ ratio serves as a critical clinical indicator for assessing immune system homeostasis. NK cells mediate non-specific cytotoxicity against tumor cells. Relevant studies have demonstrated that levels of CD3^+^ and CD4^+^ T cells, CD4^+^/CD8^+^ ratio, and NK cell activity are significantly lower in patients with advanced lung cancer compared to healthy cohorts (Wu et al., 2022b; Wu et al., 2025). The primary function of CD8^+^ cells is to eliminate pathogen-infected cells and malignant transformed cells. However, research has revealed that two CD8^+^ T-cell subtypes—CD8^+^CD28^+^ (cytotoxic T cells, Tc) and CD8^+^CD28^−^ (suppressor T cells, Ts)—exhibit antagonistic roles in immune regulation (Song et al., 2018). This study further demonstrated that compared with the chemotherapy-alone group, KAI combined with chemotherapy significantly reduced the incidence of treatment-related toxicities, including leukopenia, myelosuppression, nausea and vomiting, and gastrointestinal reactions.

As an adjuvant chemotherapeutic agent, KAI is utilized in the treatment of primary liver cancer, lung cancer, colorectal cancer, and other malignancies. This intravenous preparation is manufactured from three botanical drugs and their extracts: Panax ginseng C.A.Mey. [Araliaceae; Ginseng Radix et Rhizoma], Astragalus membranaceus (Fisch.) Bunge [Fabaceae; Astragali Radix], and Sophora flavescens Aiton [Fabaceae; Sophorae Flavescentis Radix]. Its immunomodulatory effect is not the independent action of a single component, but rather results from the synergistic interaction of multiple targets and pathways. Ultimately, it achieves the clinical outcome of “enhancing efficacy while reducing toxicity” by remodeling the tumor immune microenvironment and balancing the functions of immune cell subsets. Research has indicated that the incorporation of Astragalus membranaceus-based botanical drugs into platinum-based chemotherapy regimens reduces the incidence of chemotherapy-derived toxicities, including neutropenia, nausea, and vomiting, when compared to platinum-based chemotherapy alone. Furthermore, it is noteworthy that syndrome differentiation-based formulations of Astragalus membranaceus have demonstrated superior efficacy compared to standardized oral preparations of the botanical drug (Wang et al., 2023). In addition to reducing the incidence of adverse reactions, the combination of KAI with platinum-based chemotherapy demonstrates enhanced clinical efficacy and exhibits immunomodulatory properties within the tumor microenvironment (Li et al., 2019). UHPLC/Q-TOF-MS analysis of KAI revealed that its primary constituents include oxymatrine, astragaloside IV, and ginsenosides, among other characteristic metabolites (Li et al., 2018; Jia et al., 2020). SGK3 has been identified as a target gene of miR-367-3p. Experimental studies have confirmed that oxymatrine significantly upregulates the expression of the tumor-suppressor miR-367-3p. Under oxymatrine regulation, the enhanced miR-367-3p expression subsequently downregulates its downstream target SGK3, thereby inhibiting tumor growth and suppressing the progression of NSCLC (Yu et al., 2020). The Epidermal Growth Factor Receptor (EGFR) plays a critical role in the tumorigenesis of NSCLC (Liao et al., 2017). Experimental studies have confirmed that oxymatrine effectively suppresses both anchorage-dependent and anchorage-independent growth in NSCLC cell lines, while demonstrating no cytotoxicity toward normal pulmonary cells. Furthermore, oxymatrine significantly inhibits the activity of wild-type EGFR, exon 19 deletion-mutant EGFR, and L858R/T790M double-mutant EGFR. The compound also induces G0/G1 phase cell cycle arrest in NSCLC cells through the EGFR-Akt signaling axis. Additionally, *in vivo* experiments using xenograft mouse models have validated that oxymatrine significantly inhibits tumor growth (Li et al., 2017). Research has demonstrated that ginsenoside CK enhances the anti-proliferative, pro-apoptotic, and anti-migratory effects of gefitinib in both primary and acquired drug-resistant NSCLC. This compound suppresses the expression of HIF-1α, VEGF, FGF, and MMP-2/9, while upregulating the anti-angiogenic factor PF4 and enhancing pericellular matrix formation, ultimately promoting vascular structure normalization (Song et al., 2024). Studies in murine models have revealed that ginsenoside Rh2 promotes the infiltration of CD4^+^ and CD8^+^ T lymphocytes into tumor tissues, thereby enhancing anti-tumor efficacy (Wang et al., 2017). Research has demonstrated that high-dose astragaloside IV significantly inhibits NSCLC cell proliferation, while low-dose treatments show no apparent cytotoxicity. Furthermore, the combination of astragaloside IV with cisplatin enhances chemosensitivity in NSCLC cells through suppression of B7-H3 expression at both transcriptional and protein levels (He et al., 2016). Research has further demonstrated that the combination of astragaloside IV with anti-PD-1 therapy inactivates both PI3K/Akt and ERK signaling pathways, suppresses tumor cell proliferation, and induces apoptosis. This combined treatment promotes M1 macrophage polarization and T-cell activation, resulting in significant reduction of tumor volume and weight in LLC tumor-bearing mice (Wu et al., 2024). Furthermore, studies indicate that astragaloside III enhances the anti-tumor response of natural killer (NK) cells by upregulating the expression of NKG2D and IFN-γ (Chen et al., 2019). The aforementioned components collectively regulate immune function through complementary pathways, which directly correlates with the findings in this study, namely, the increased percentages of CD3^+^ and CD4^+^ T cells and enhanced NK cell activity observed in the combination therapy group. This provides a theoretical foundation for KAI’s role in improving immune function.

## 5 Study strengths and limitations

The strengths of this study lie in its strict adherence to the PRISMA guidelines and the prospective registration of the study protocol on the PROSPERO platform, ensuring methodological rigor. Through systematic searches of Chinese and English databases, we included 14 randomized controlled trials with a total sample size of 1,214 patients. Data extraction and quality assessment were independently performed by two investigators with cross-verification, effectively minimizing the risk of subjective bias. Our comprehensive analysis not only evaluated clinical efficacy and core immune function parameters but also systematically assessed multiple chemotherapy-related adverse reactions. Furthermore, we validated the robustness of the primary outcomes through subgroup analyses, sensitivity analyses, and trial sequential analysis, thereby enhancing the reliability of our conclusions.

This study has several limitations. All studies included in this analysis were conducted in China, indicating a significant geographical limitation. On one hand, differences in genetic background, dietary structure, and the principles of Traditional Chinese Medicine diagnosis and treatment based on pattern differentiation between the Chinese population and those in other countries or regions may lead to racial heterogeneity in the therapeutic response to KAI. On the other hand, while the selection of chemotherapy regimens and standards for supportive care for NSCLC within the Chinese healthcare system align with international guidelines to some extent, distinct regional characteristics persist, which may influence the assessment of adverse event incidence rates. Most studies provided inadequate reporting on key methodological aspects such as random sequence generation, allocation concealment, and blinding implementation, potentially introducing performance and detection biases. The evidence for certain critical outcomes, including NK cell parameters, was limited by the small number of included studies. Furthermore, several immune indicators demonstrated substantial statistical heterogeneity; although random-effects models were employed and subgroup analyses conducted, the underlying clinical heterogeneity could not be fully elucidated. Therefore, the generalizability of the conclusions from this study requires cautious interpretation. Future research should involve multicenter, cross-regional randomized controlled trials that enroll patients of diverse ethnicities and from varying healthcare environments to further validate the efficacy and safety of KAI combined with chemotherapy.

## 6 Conclusion

The combination of KAI with chemotherapy for advanced non-small cell lung cancer demonstrates immunomodulatory effects by enhancing peripheral blood CD3^+^, CD4^+^, CD4^+^/CD8^+^ ratios and NK cell percentages, while reducing tumor marker levels including CEA and VEGF. This integrated approach achieves the clinical effect of “enhancing efficacy and reducing toxicity by improving objective response rates and simultaneously decreasing chemotherapy-related adverse reactions such as leukopenia, myelosuppression, nausea, and vomiting, thereby holding significant importance for patients’ quality of life. However, the strength of evidence remains constrained by methodological limitations in included studies, including generally low quality, high population homogeneity, and absence of long-term survival data. Future investigations should prioritize multicenter, large-sample, double-blind randomized controlled trials to optimize KAI dosage regimens and treatment duration, while further validating its long-term efficacy and applicability across diverse ethnic populations, thereby providing higher-level evidence for its standardized application in advanced NSCLC treatment.

## Data Availability

The original contributions presented in the study are included in the article/Supplementary Material, further inquiries can be directed to the corresponding authors.

## References

[B1] Bade B. C. Dela Cruz C. S. (2020). Lung cancer 2020: epidemiology, etiology, and prevention. Clin. Chest Med. 41, 1–24. 10.1016/j.ccm.2019.10.001 32008623

[B2] Bray F. Laversanne M. Sung H. Ferlay J. Siegel R. L. Soerjomataram I. (2024). Global cancer statistics 2022: GLOBOCAN estimates of incidence and mortality worldwide for 36 cancers in 185 countries. CA. Cancer J. Clin. 74, 229–263. 10.3322/caac.21834 38572751

[B3] Chen L. (2014). Effect of kang’ai injection on immune function and quality of life in patients with mid-advanced non-small cell lung cancer during chemotherapy. Guid. J. Traditional Chin. Med. Pharm. 20, 29–34. 10.13862/j.cnki.cn43-1446/r.2014.05.010

[B4] Chen X. Chen X. Gao J. Yang H. Duan Y. Feng Y. (2019). Astragaloside III enhances anti-tumor response of NK cells by elevating NKG2D and IFN-γ. Front. Pharmacol. 10, 898. 10.3389/fphar.2019.00898 31456687 PMC6701288

[B5] Cheng Z. Liu Y. Ma M. Sun S. Ma Z. Wang Y. (2022). Effect of kang’ai injection combined with conventional chemotherapy on immune function and safety in patients with advanced non-small cell lung cancer. Medicine 28, 21–25. 10.1186/s10020-022-00448-x 35183103 PMC8858482

[B6] De Ruysscher D. Faivre-Finn C. Nackaerts K. Jordan K. Arends J. Douillard J. Y. (2020). Recommendation for supportive care in patients receiving concurrent chemotherapy and radiotherapy for lung cancer. Ann. Oncol. Off. J. Eur. Soc. Med. Oncol. 31, 41–49. 10.1016/j.annonc.2019.10.003 31912794

[B7] Dong H. Wang C. Gong Q. (2019). Effect of kang’ai injection as an adjuvant to chemotherapy on immune function and tumor markers in elderly patients with mid-advanced stage non-small cell lung cancer. Chin. J. Gerontology 39, 52–55. 10.3969/j.issn.1005-9202

[B8] Ernani V. Steuer C. E. Jahanzeb M. (2017). The end of nihilism: systemic therapy of advanced non-small cell lung cancer. Annu. Rev. Med. 68, 153–168. 10.1146/annurev-med-042915-102442 27618751

[B9] Gao M. (2018). Effects of kang’ai injection combined with PC chemotherapy on KPS score and serum T cell subsets, CEA levels in patients with advanced non-small cell lung cancer. Available online at: https://rs.yiigle.com/cmaid/1036100 (Accessed October 3, 2025).

[B10] Gao W. Zhang K. (2023). Network meta-analysis of 8 types of traditional Chinese medicine injection combined with chemotherapy in colorectal cancer treatment. J. Cancer Res. Clin. Oncol. 149, 9823–9838. 10.1007/s00432-023-04892-y 37246189 PMC11797444

[B11] Gong F. Zhou L. liu X. (2024). Effect of kang’ai injection combined with bevacizumab and chemotherapy on immune function and serum tumor markers in patients with non-squamous non-small cell lung cancer. Prim. Care Forum 28, 130–147. 10.19435/j.1672-1721.2024.13.041

[B12] Greiber T. (2019). Implementation of the nagoya protocol in the european union and in Germany. Phytomed. Int. J. Phytother. Phytopharm. 53, 313–318. 10.1016/j.phymed.2018.10.020 30392747

[B13] He C.-S. Liu Y.-C. Xu Z.-P. Dai P.-C. Chen X.-W. Jin D.-H. (2016). Astragaloside IV enhances cisplatin chemosensitivity in non-small cell lung cancer cells through inhibition of B7-H3. Cell. Physiol. Biochem. Int. J. Exp. Cell. Physiol. biochem. Pharmacol. 40, 1221–1229. 10.1159/000453175 27960166

[B14] Hoy H. Lynch T. Beck M. (2019). Surgical treatment of lung cancer. Crit. Care Nurs. Clin. North Am. 31, 303–313. 10.1016/j.cnc.2019.05.002 31351552

[B15] Jia M. Zhang B. Qi Y. Yang J. Yao Z. Qin Z. (2020). UHPLC coupled with mass spectrometry and chemometric analysis of kang-ai injection based on the chemical characterization, simultaneous quantification, and relative quantification of 47 herbal alkaloids and saponins. J. Sep. Sci. 43, 2539–2549. 10.1002/jssc.201900878 32250549

[B16] Jiang H. Zheng K. Tong l (2018). Effect of Kangai injection combined with GP +chemotherapy in the treatment of patients with advanced non-small cell lung cancer and its influence on quality of life. Chin. J. Prim. Med. Pharma 25, 1447–1451. 10.3760/cma.j.issn.1008-6706.2018.11.023

[B17] Lechner A. Schlößer H. Rothschild S. I. Thelen M. Reuter S. Zentis P. (2017). Characterization of tumor-associated T-lymphocyte subsets and immune checkpoint molecules in head and neck squamous cell carcinoma. Oncotarget 8, 44418–44433. 10.18632/oncotarget.17901 28574843 PMC5546490

[B18] Li W. Yu X. Tan S. Liu W. Zhou L. Liu H. (2017). Oxymatrine inhibits non–small cell lung cancer via suppression of EGFR signaling pathway. Cancer Med. 7, 208–218. 10.1002/cam4.1269 29239135 PMC5773973

[B19] Li P. Zhao P. Liu W. Jiang Y. Wang W. Bao L. (2018). Determination of common ginsenosides in kang’ai injection by aqueous two-phase extraction with deep eutectic solvents and HPLC-UV/DAD. Microchem. J. 137, 302–308. 10.1016/j.microc.2017.11.007

[B20] Li H. Ji Y. Zhang S. Gao Z. Hu C. Jiang R. (2019). Kangai injection combined with platinum-based chemotherapy for the treatment of stage III/IV non-small cell lung cancer: a meta-analysis and systematic review of 35 randomized controlled trials. J. Cancer 10, 5283–5298. 10.7150/jca.31928 31602279 PMC6775612

[B21] Li Z. Feiyue Z. Gaofeng L. (2021). Traditional chinese medicine and lung cancer--from theory to practice. Biomed. Pharmacother. = Biomed. Pharmacother. 137, 111381. 10.1016/j.biopha.2021.111381 33601147

[B22] Li S. de Camargo Correia G. S. Wang J. Manochakian R. Zhao Y. Lou Y. (2023). Emerging targeted therapies in advanced non-small-cell lung cancer. Cancers 15, 2899. 10.3390/cancers15112899 37296863 PMC10251928

[B23] Liao B.-C. Lin C.-C. Lee J.-H. Yang J. C.-H. (2017). Optimal management of EGFR-mutant non-small cell lung cancer with disease progression on first-line tyrosine kinase inhibitor therapy. Lung Cancer (amst. Neth.) 110, 7–13. 10.1016/j.lungcan.2017.05.009 28676222

[B24] Ma M. (2017). Clinical Study on kang’ai injection combined with TP chemotherapy in the treatment of mid-advanced stage non-small cell lung cancer. Asia-Pacific Tradit. Med. 13, 163–165. 10.11954/ytctyy.201720067

[B25] Meng X. Gao Y. Yang L. Jing H. Teng F. Huang Z. (2019). Immune microenvironment differences between squamous and non-squamous non-small-cell lung cancer and their influence on the prognosis. Clin. Lung Cancer 20, 48–58. 10.1016/j.cllc.2018.09.012 30341017

[B26] Nie X. Xiong H. (2025). Effect of Kang′ ai injection combined with Albumin Paclitaxel and Cisplatin in the treatment of non-small cell lung cancer. China Contemp. Med. 32, 55–65. 10.3969/j.issn.1674-4721.2025.20.12

[B27] Page M. J. McKenzie J. E. Bossuyt P. M. Boutron I. Hoffmann T. C. Mulrow C. D. (2021). The PRISMA 2020 statement: an updated guideline for reporting systematic reviews. Syst. Rev. 10, 89. 10.1186/s13643-021-01626-4 33781348 PMC8008539

[B28] Peled M. Onn A. Herbst R. S. (2019). Tumor-infiltrating lymphocytes-location for prognostic evaluation. Clin. Cancer Res. Off. J. Am. Assoc. Cancer Res. 25, 1449–1451. 10.1158/1078-0432.CCR-18-3803 30567833

[B30] Song Q. Ren J. Zhou X. Wang X. Song G. Hobeika A. (2018). Circulating CD8+CD28-suppressor T cells tied to poorer prognosis among metastatic breast cancer patients receiving adoptive T-cell therapy: a cohort study. Cytotherapy 20, 126–133. 10.1016/j.jcyt.2017.08.018 28988693

[B31] Song X. Wang L. Cai P. Xu Y. Liu Q. Fan D. (2024). Synergistic anticancer effects of ginsenoside CK and gefitinib against gefitinib-resistant NSCLC by regulating the balance of angiogenic factors through HIF-1α/VEGF. Toxicol. Appl. Pharmacol. 486, 116938. 10.1016/j.taap.2024.116938 38642809

[B32] Sun C. Dong F. Xiao T. Gao W. (2021). Efficacy and safety of chinese patent medicine (kang-ai injection) as an adjuvant in the treatment of patients with hepatocellular carcinoma: a meta-analysis. Pharm. Biol. 59, 472–483. 10.1080/13880209.2021.1915340 33905666 PMC8081330

[B33] Tang C. (2019). Clinical efficacy of Kangai injection combined with conventional chemotherapy and Bevacizumab in the treatment of non-squamous non-small cell lung cancer and effect on the immune function. Basic Clin. Oncol. 32, 277–282. 10.3969/j.issn.1673-5412

[B34] Terracciano L. Brozek J. Compalati E. Schünemann H. (2010). GRADE system: new paradigm. Curr. Opin. Allergy Clin. Immunol. 10, 377–383. 10.1097/ACI.0b013e32833c148b 20610980

[B35] Wang Z. (2017). Observation on the efficacy of first-line platinum-based chemotherapy combined with kang’ai injection in the treatment of advanced non-small cell lung cancer. Clin. Med. 37, 101–102. 10.19528/j.issn.1003-3548.2017.10.051

[B36] Wang M. Yan S.-J. Zhang H.-T. Li N. Liu T. Zhang Y.-L. (2017). Ginsenoside Rh2 enhances the antitumor immunological response of a melanoma mice model. Oncol. Lett. 13, 681–685. 10.3892/ol.2016.5490 28356946 PMC5351349

[B37] Wang Q. Jiao L. Wang S. Chen P. Bi L. Zhou D. (2020). Adjuvant chemotherapy with Chinese herbal medicine formulas versus placebo in patients with lung adenocarcinoma after radical surgery: a multicenter, randomized, double-blind, placebo-controlled trial. Biol. Proced. Online 22, 5. 10.1186/s12575-020-00117-5 32140080 PMC7049384

[B38] Wang R. Deng Z. Zhu Z. Wang J. Yang X. Xu M. (2023). Kaempferol promotes non-small cell lung cancer cell autophagy via restricting met pathway. Phytomed. Int. J. Phytother. Phytopharm. 121, 155090. 10.1016/j.phymed.2023.155090 37738907

[B39] Wang J. Xi H. Chen X. Xin Y. Wei F. (2025). Efficacy and safety of Chinese medicine injection combined with concurrent chemoradiotherapy in the treatment of esophageal cancer: a bayesian network meta-analysis. Front. Med. 12, 1643598. 10.3389/fmed.2025.1643598 41164166 PMC12558960

[B40] Wu Y. (2021). Clinical efficacy of kang’ai injection combined with chemotherapy in the treatment of advanced non-small cell lung cancer. Chin J Clin. Ration. Drug Use 14, 76–78. 10.15887/j.cnki.13-1389/r.2021.18.027

[B41] Wu X. Chen C. Xu H. (2022a). Effects of Kang′ ai injection on VEGF and immune function in elderly patients with advanced NSCLC treated with cisplatin -containing chemotherapy. China Mod. Dr. 60, 8–24. 10.3969/j.issn.1673-9701.2022.11.zwkjzlml-yyws202211003

[B42] Wu Z. Zheng Y. Sheng J. Han Y. Yang Y. Pan H. (2022b). CD3+CD4-CD8- (Double-Negative) T cells in inflammation, immune disorders and cancer. Front. Immunol. 13, 816005. 10.3389/fimmu.2022.816005 35222392 PMC8866817

[B43] Wu T. Wu S. Gao H. Liu H. Feng J. Yin G. (2024). Astragaloside IV augments anti-PD-1 therapy to suppress tumor growth in lung cancer by remodeling the tumor microenvironment. Eur. J. Histochem, EJH 68, 4098. 10.4081/ejh.2024.4098 39440587 PMC11558310

[B44] Wu M. Wang X. Zhu J. Wang C. Gu M. Wang B. (2025). Clinical significance of peripheral blood lymphocyte subsets and cytokine profiles in patients with respiratory syncytial virus infection. Microbes Infect., 105583. 10.1016/j.micinf.2025.105583 41177515

[B45] Xing H. (2020). Effect observation Kang-ai injection combined with chemotherapy in treatment of advanced non-small cell lung cancer. World Latest Medicne Inf. Electron. Version 20, 26–27. 10.19613/j.cnki.1671-3141.2020.2.013

[B46] Xue G. Guo H. Bai J. (2022). Effect of kang’ai injection combined with GP chemotherapy regimen in the treatment of elderly patients with advanced non-small cell lung cancer and its influences on immune function, VEGF, CEA and NSE levels. Clin. Res. Pract. 7, 143–149. 10.19347/j.cnki.2096-1413.202201036

[B47] Yu Q. Luo J. Zhang J. Chen Y. Chen K. Lin J. (2020). Oxymatrine inhibits the development of non-small cell lung cancer through miR-367-3p upregulation and target gene SGK3 downregulation. Am. J. Transl. Res. 12, 5538–5550. Available online at: https://pubmed.ncbi.nlm.nih.gov/33042436/. 33042436 PMC7540135

[B48] Yuan Y.-F. Chen X.-Y. Liu B.-X. Liu X. Li R.-H. Qi J.-L. (2017). Simultaneous determination of 11 components in Kang’ai Injection by UPLC-MS/MS. Chin. Tradit. Herb. Drugs 48, 2660–2665. 10.7501/j.issn.0253-2670.2017.13.012

[B49] Zraik I. M. Heß-Busch Y. (2021). Management of chemotherapy side effects and their long-term sequelae. Urol. Ausg, A 60, 862–871. 10.1007/s00120-021-01569-7 34185118

