# Supplementary material for: Effects of Kang’ai injection combined with chemotherapy on immune function in advanced non-small cell lung cancer: a meta-analysis
Source: Front Pharmacol. 2026 Jan 23;17:1743226. doi: 10.3389/fphar.2026.1743226 (PMC12876135; doi:10.3389/fphar.2026.1743226)
Supplement: Supplementary file 1 [file Table1.docx]

***Supplementary Materials***

**Table of contents**

[Supplementary Material 1: Search strategies 1](#_Toc17908)

[Table 1. Search strategy for PubMed database. 1](#_Toc4079)

[Table 2. Search strategy for EMBASE database. 1](#_Toc32409)

[Table 3. Search strategy for Web of Science database. 1](#_Toc25592)

[Table 4. Search strategy for Cochrane database. 1](#_Toc6711)

[Table 5. Search strategy for China National Knowledge Infrastructure/CNKI database. 1](#_Toc5397)

[Table 6. Search strategy for VIP's Chinese Science and Technology Journal Database/VIP. 2](#_Toc6554)

[Table 7. Search strategy for Wanfang Database. 2](#_Toc732)

[Table 8. Search strategy for China Biology Medicine/CBM. 2](#_Toc8212)

[Table 9. Search strategy for Chinese Medical Journals Database (CMJD). 2](#_Toc17697)

**Supplementary Material 1:** Search strategies

Table 1. Search strategy for PubMed database.

| **ID** | **Search** | **Hits** |
| --- | --- | --- |
| #1 | (Neoplasm [Mesh] OR Lung Neoplasm [Mesh] OR Pulmonary Neoplasms OR Lung Cancer OR NSCLC OR Non-small Cell Lung Cancer) | 4281363 |
| #2 | (Chemotherapy OR Chemotherapeutics OR Chemical therapy)[All Fields] | 4531783 |
| #3 | (Kangai injection OR Kangai OR Kang’ai)[All Fieldst] | 335 |
| #4 | #1 AND #2 AND #3 | 75 |
| **Last Run Date: 09/15/2025** | | |

Table 2. Search strategy for EMBASE database.

| **ID** | **Search** | **Hits** |
| --- | --- | --- |
| #1 | ' Non-small Cell Lung Canceri'':ab,kw,ti | 275116 |
| #2 | Chemotherapyi':ab,kw,ti | 178613 |
| #3 | 'Kangai injection':ab,kw,ti OR 'Kang’a':ab,kw,ti | 816 |
| #4 | #1 AND #2 AND #3 | 10 |
| **Last Run Date: 09/15/2025** | | |

Table 3. Search strategy for Web of Science database.

| **ID** | **Search** | **Hits** |
| --- | --- | --- |
| #1 | TS=((Neoplasm [Mesh] OR Lung Neoplasm [Mesh] OR Pulmonary Neoplasms OR Lung Cancer OR NSCLC OR Non-small Cell Lung Cancer) ) | 415439 |
| #2 | TS=(Chemotherapy OR Chemotherapeutics OR Chemical therapy) | 566630 |
| #3 | TS= (Kangai injection OR Kangai OR Kang’ai) | 116 |
| #4 | #1 AND #2 AND #3 | 15 |
| **Last Run Date: 09/15/2025** | | |

Table 4. Search strategy for Cochrane database.

| **ID** | **Search** | **Hits** |
| --- | --- | --- |
| #1 | （Kangai injection OR Kangai OR Kang’ai）：:ti,ab,kw | 70 |
| #2 | (Neoplasm OR Lung Neoplasm OR Pulmonary Neoplasms OR Lung Cancer OR NSCLC OR Non-small Cell Lung Cancer):ti,ab,kw | 864 |
| #3 | (Chemotherapy OR Chemotherapeutics OR Chemical therapy):ti,ab,kw | 933 |
| #4 | #1 AND #2 AND #3 | 24 |
| **Last Run Date: 09/15/2025** | | |

Table 5. Search strategy for China National Knowledge Infrastructure/CNKI database.

| **ID** | **Search** | **Hits** |
| --- | --- | --- |
| #1 | (SU%=' Kangai injection i) OR(SU%=' Kangai ) OR(SU%=' Kang’ai) OR(KY=' Kangai injection ) OR(KY=' Kangai ) OR(KY=' Kang’ai) OR(FT=' Kangai injection ) OR(FT=' Kangai ) OR(FT=' Kang’ai) | 5585 |
| #2 | (TI='Chemotherapy) | 143800 |
| #3 | (SU%=' Non-small Cell Lung Cancer) OR(KY=' Non-small Cell Lung Cancer) OR(KY=' Non-small Cell Lung Cancer ) | 66300 |
| #4 | #1 AND #2 AND #3 | 137 |
| **Last Run Date: 09/15/2025** | | |

Table 6. Search strategy for VIP's Chinese Science and Technology Journal Database/VIP.

| **ID** | **Search** | **Hits** |
| --- | --- | --- |
| #1 | (M="Kangai injection" ) OR (T="Kangai injection") OR (R="Kangai injection" ) | 3708 |
| #2 | (M="Chemotherapy" ) OR (T="Chemotherapy") OR (R="Chemotherapy" ) | 47083 |
| #3 | (M="Non-small Cell Lung Cancer" ) OR (T="Non-small Cell Lung Cancer") OR (R="Non-small Cell Lung Cancer" ) | 534414 |
| #4 | #1 AND #2 AND #3 | 101 |
| **Last Run Date: 09/15/2025** | | |

Table 7. Search strategy for Wanfang Database.

| **ID** | **Search** | **Hits** |
| --- | --- | --- |
| #1 | Subject:("Kangai injection") OR Title OR Keywords:("Kangai injection" ) OR Abstract:("Kangai injection") | 196 |
| #2 | Subject:("Chemotherapy") OR Title OR Keywords:("Chemotherapy" ) OR Abstract:("Chemotherapy") | 428832 |
| #3 | Subject:("Non-small Cell Lung Cancer") OR Title OR Keywords:("Non-small Cell Lung Cancer" ) OR Abstract:("Non-small Cell Lung Cancer") | 81783 |
| #4 | #1 AND #2 AND #3 | 179 |
| **Last Run Date: 09/15/2025** | | |

Table 8. Search strategy for China Biology Medicine/CBM.

| **ID** | **Search** | **Hits** |
| --- | --- | --- |
| #1 | "Kangai injection"[Chinese Title: Intelligent] OR "Kangai injection"[Abstract: Intelligent] | 867 |
| #2 | "Chemotherapy"[Chinese Title: Intelligent] OR "Chemotherapy"[Abstract: Intelligent] | 270540 |
| #3 | "Non-small Cell Lung Cancer"[Chinese Title: Intelligent] OR "Non-small Cell Lung Cancer"[Abstract: Intelligent] | 49995 |
| #4 | #1 AND #2 AND #3 | 142 |
| **Last Run Date: 09/15/2025** | | |

Table 9. Search strategy for Chinese Medical Journals Database (CMJD).

| **ID** | **Search** | **Hits** |
| --- | --- | --- |
| #1 | TI="Kangai injection" OR KW="Kangai injection" | 26 |
| #2 | TI="Chemotherapy" OR KW="Chemotherapy" | 38066 |
| #3 | TI="Non-small Cell Lung Cancer" OR KW="Non-small Cell Lung Cancer" | 6689 |
| #4 | #1 AND #2 AND #3 | 9 |
| **Last Run Date: 09/15/2025** | | |
